# Assessment of Irrigation Efficiency by Coupling Remote Sensing and Ground-Based Data: Case Study of Sprinkler Irrigation of Alfalfa in the Saratovskoye Zavolgie Region of Russia

**DOI:** 10.3390/s23052601

**Published:** 2023-02-26

**Authors:** Anatoly Mikhailovich Zeyliger, Olga Sergeevna Ermolaeva, Viktor Vladimirovich Pchelkin

**Affiliations:** Department of Applied Informatics, Institute of Economics and Management in Agribusiness, Russian State Agrarian University—Moscow Timiryazev Agricultural Academy, Timiryazevskaya Str., 49, 127550 Moscow, Russia

**Keywords:** irrigation, irrigation efficiency, water productivity, irrigation water use efficiency, weather water demand, alfalfa crop, evapotranspiration, transpiration, sensor-based monitoring, SEBAL model, MODIS data

## Abstract

Nowadays, the leading role of data from sensors to monitor crop irrigation practices is indisputable. The combination of ground and space monitoring data and agrohydrological modeling made it possible to evaluate the effectiveness of crop irrigation. This paper presents some additions to recently published results of field study at the territory of the Privolzhskaya irrigation system located on the left bank of the Volga in the Russian Federation, during the growing season of 2012. Data were obtained for 19 crops of irrigated alfalfa during the second year of their growing period. Irrigation water applications to these crops was carried out by the center pivot sprinklers. The actual crop evapotranspiration and its components being derived with the SEBAL model from MODIS satellite images data. As a result, a time series of daily values of evapotranspiration and transpiration were obtained for the area occupied by each of these crops. To assess the effectiveness of irrigation of alfalfa crops, six indicators were used based on the use of data on yield, irrigation depth, actual evapotranspiration, transpiration and basal evaporation deficit. The series of indicators estimating irrigation effectiveness were analyzed and ranked. The obtained rank values were used to analyze the similarity and non-similarity of indicators of irrigation effectiveness of alfalfa crops. As a result of this analysis, the opportunity to assess irrigation effectiveness with the help of data from ground and space-based sensors was proved.

## 1. Introduction

### 1.1. Actual Situation in Agricultural Irrigation Sector of RF

In recent years, the territories located in the south-east of the Russian Federation are regularly exposed to periodic droughts [1,2]. Such natural-climatic phenomena affect significant areas of agricultural production in different regions, giving rise to serious problems in obtaining secure yields [3,4,5]. Moreover, the deficiency of water resources is fraught with risks that come from under-obtaining secure high yields of irrigated agricultural crops [6,7,8,9]. The use of traditional irrigation technologies, in particular, in regions with a shortage of water resources, does not create the necessary conditions for the rational use of irrigation water [10]. It is complicated by the lack of systematic and objective, quantitative assessment of the use of water for the irrigation of agricultural crops. As a result, in many regions having irrigated agriculture there is a lack of target values and criteria aimed at the development and perfection of the technologies of irrigation that must provide a decrease in specific indices of irrigation per the yield unity [11]. The lack of such criteria does not allow the formation of substantiated policy directed at the provision of higher crop yields with less expenditure of irrigation water, and at spreading irrigation over the new areas due to the saved volumes of water resources.

In recent years, a number of agricultural regions in the south and south-east of the European part of the Russian Federation are subjected to the situation of the deficiency of the surface water resources that are necessary, in particular, for the extensive development of irrigated agriculture [12]. In many respects it is connected with the historically formed practice of irrigation performance when part of the river run-off was taken to be used on the irrigation systems, built in the second part of the 20th century in the south-east part of the European part of RF. Under classification [13], these irrigation systems that are mainly performed by sprinkling with the relatively short period of irrigation (3–4 months). These systems are pumping water from water storages located on the riversides of densely populated rivers Volga and Don. For both of these water basins some significant fluctuations of meteorological factors and conditions were observed over resent years [14] and projected for the near future [15], which caused and might still cause high risk of water resource deficits.

### 1.2. Quantification of the Irrigation Efficiency

Currently, in the practice of analyzing the efficiency of irrigation water use in irrigated agriculture, a number of indicators (indicators) are used [16,17], none of which is universal due to their inherent limitations [18,19,20,21,22,23].

Methods that increase efficiency of the use of irrigation water give rise to the economic efficiency and ecological stability of irrigated agriculture; this is not necessarily realized due to the reduction in the volumes of irrigation water. For the appraisal of the efficiency of such methods different models assessing how the agricultural crops use water stored in the root layer of soil cover have been developed, and are being developed further [16,24,25,26,27,28,29].

### 1.3. Indicators of the Efficiency of the Irrigation Water Use by the Agricultural Crops

The choice of the efficiency indicators from a relatively large list [13,17,30,31,32] is not an easy task due to the presence of a high variety of infrastructures [13,32]. The further analysis of the values of the indicators obtained has a prior significance for the rational improvements, aimed at the raising of the overall efficiency to the attainable level [16,24,25,33,34].

Variety of indicators of the efficiency of the irrigation water use can be contingently classified into the following groups:

1. Indicators of water efficiency of irrigation systems at the river basin level [17,35,36,37];
2. Irrigation water efficiency indicators at the irrigation system level [38,39];
3. Indicators of irrigation water efficiency at the level of irrigated agrophytocenosis [38,39,40].

The use of indicators of the first group allows us to assess how the external factors will influence the results of realization of irrigation at the levels, directly connected with the agro-hydrological conditions and factors. This is achieved using the relevant values of the efficiency of irrigation, connected with the results of agricultural production at the corresponding agro-hydrological levels on the basis of the available data on control and monitoring. Therefore, part of such indicators allows us to gain relevant assessments for the characteristics of the irrigation system as a whole. As a result, they can be used both for the assessment of the current state of operational characteristics of the irrigation systems and of its impact on the social-economic indexes of the corresponding region, as well as for the assessment of the potential abilities of such systems towards improving their efficiency.

A set of indicators of the second and of the third groups, used directly for the analysis of the efficiency of realization of irrigation regimes, is mostly based on the indicators that we are grouping in two categories: Water Use Efficiency (WUE) [10,18,41,42,43,44,45,46,47,48,49,50,51,52,53] and Water Productivity (WP) [17,38]. Often both groups of indicators are used interchangeably, although they give assessments from somewhat different positions.

It is well known that the key requirement for increasing the efficiency of the use of irrigation water for irrigation of agrophytocenoses is the compliance of the created water regime with the actual needs of agricultural crops. At the same time, it is also well known that in addition to the correct planning of the timing and volume of irrigation of industrial crops, an appropriate implementation is also necessary, minimizing possible inconsistencies; one of the leading places of which is given to unproductive losses of irrigation water due to evaporation from the soil surface, as well as soil and ground stock.

The traditional techniques available for a posteriori assessment of irrigation results using the basic integral parameters—the yield obtained, and the volume of irrigation water used. However, due to the integral nature of these parameters it is often insufficient for a deeper analysis, in terms of not only the timing and volume of irrigation but also the parameters characterizing the use of irrigation water by agrophytocenosis throughout their growth and development. In this regard, the use of the water balance component of the root zone of soil cover of agrophytocenosis characteristics is a promising challenge to the development for quantification of irrigation result analyses. One of the major techniques available now is an estimation of actual evaporation (ETa) and its components done with a model using data from space-based sensors [54,55,56,57,58,59,60,61].

In this study, we compared estimates of irrigation efficiency based on traditional integral indicators with similar estimates based on indicators obtained using data from space-based sensors for 19 simultaneously growing irrigated alfalfa crops in the territories of three neighboring study sites. At the same time, our goal was to demonstrate the performance of the indicators second group, as well as their higher sensitivity when conducting an appropriate analysis.

## 2. Materials and Methods

### 2.1. Study Area

The study was conducted in Saratovskoye Zavolzhie located between the left bank of the middle part of Volga River, in the south-east of the European part of RF and border of Kazakhstan. This region of risky agriculture due to quite frequent drought is characterized by a continental arid climate with cold winters and hot summers [62]. The duration of the frost-free period is approximately 150–160 days per year. The sum of active temperatures is 2600–2800 °C, which allows the cultivation of a wide range of agricultural crops. The average annual precipitation in this area is approximately 350–450 mm, the majority of which occurs during winter and spring [62]. The typical soil cover mostly consists of dark-chestnut soils. Among them there occur spots of chestnut-meadow and solonetz-chestnut soils. Groundwater mostly lies at a depth below 10 m, although in some irrigated areas it occurs at depths of 2–3 m. A shallow water depth developed by irrigation water leakage from water supply canals and irrigated fields is leading a number of sites to soil salinization and bogging.

**Figure 1:**
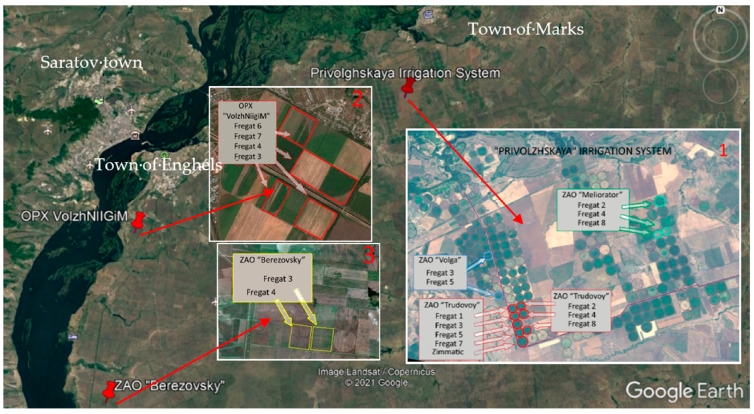
Three study sites belonging to the territories of: (1) Privolzhskaya Irrigation System; (2) Experimental farm of the All-Russian Scientific Research Institute of Hydraulic Engineering and Land Reclamation (VolgNIIGiM); (3) “Berezovsky” agricultural farm (copied from Google Earth).

The locations of the 19 studied irrigated alfalfa crops of the 2nd year of vegetation during 2012 were confined to three neighboring study sites inside of the region with developed industrial irrigation, are shown in Figure 1.

Irrigation at three study sites of alfalfa crops was carried out by center pivot irrigation machines of the Fregat and Zimmatic types. At the same time, to the territory of the 1st study site, irrigation water was transported through the water supply infrastructure of the Privolzhskaya irrigation system to 13 alfalfa crops owned by three agricultural farms: (1) “Volga” agricultural farm with 13 alfalfa crops; (2) “Trudovoy” agricultural farm with 8 alfalfa crops; (3) “Meliorator” agricultural farm with 3 alfalfa crops. On the territory of the 2nd study site, irrigation water to 3 alfalfa crops, which belonged to VolzhNIIGiM, was transported through the water supply infrastructure of the Saratov irrigation system. On the territory of the 3rd study site owned by the “Berezovsky” agricultural farm, irrigation water to 2 alfalfa crops was transported through a pipeline into which it was forced by a floating pumping station located directly on the river Volga.

#### Weather Conditions, Harvest and Irrigation Timing

In 2012 spring snow melting took place at the beginning of April. After that, the surface layer of air started to warm up quickly enough. This gave an impetus to the development of the alfalfa as well as transpiration flow. In the research area at the end of April, the air drought was observed owing to the movement of dry heated air masses from the east. A similar situation was observed in the second 10 days (decade) of May. This caused desiccation of the soil upper layer and deceleration of vegetation of alfalfa crops. Water storage in the root zone of the soil cover, needed for crops, was replenished owing to slight rains in the middle and at the end of May, and due to the 1st irrigation applied in a number of crops including alfalfa.

The 1st harvest of alfalfa at the end of the first decade of June was followed by the water irrigation application. In the middle of June, before the 2nd harvest, the rain front passed with the following precipitation that reduced the need for irrigation in this period. The 3rd irrigation application was performed after the 2nd harvest in the 1st decade of July. The following hot period resulted in quick expenditure of soil water storage in the root zone of soil cover, in spite of a small precipitation fall at the end of the second decade of July. As a result, vegetation development started to slow down. The 4th irrigation application was performed in the period from the end of July to the beginning of August. At the beginning of the second decade of August the 3rd harvest was performed following water irrigation application as well. After that the rain front passed, which coincided with heavy precipitation.

### 2.2. Ground and Satellite Data Set Collection

For the assessment of irrigation effectiveness based on using the ground and remote sensing for each irrigated alfalfa crop, the dataset of the appropriate scale was collected. Each dataset includes timing and yield of three harvestings, irrigation water applications, meteorological parameters, information gathered during visual scouting, as well as the time series of daily values of *ETa*, *Tr* and *Ev*, calculated by the SEBAL model based on the results of space monitoring by the MODIS sensor (or Moderate Resolution Imaging Spectroradiometer).

#### 2.2.1. Meteorological Data Sets

**Figure 2:**
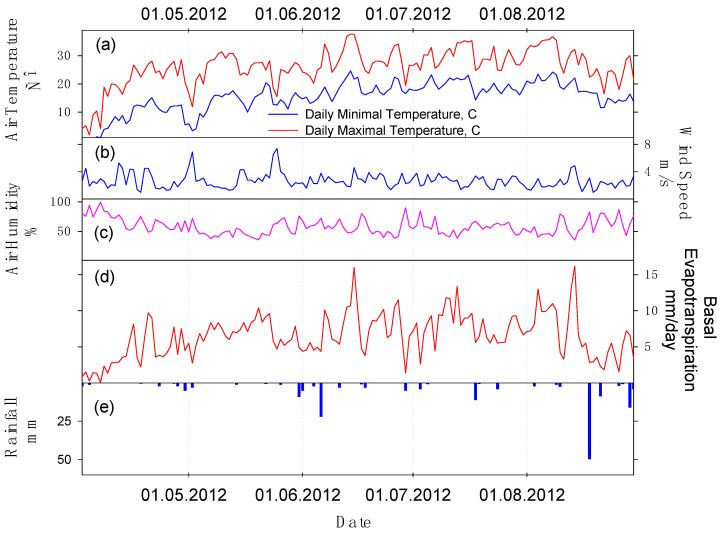
Agrometeorological characteristics of Marks town meteorological station: (**a**) air temperature; (**b**) wind speed; (**c**) air humidity; (**d**) basal evapotranspiration; (**e**) rainfall.

Dynamics of the temperature and humidity of air and wind speed give a satisfactory display of the influence of meteorological conditions of the research area shown in the Figure 2.

Meteorological data of the meteorological station of town Marks, and also radiometric data of the temperature measurements by the radiometer MODIS/EOS Terra and Aqua, were used as the input parameters for calculation of the layers of the daily flows of *ETa* and its components. In order to gain the daily values of these flows from the areas of each studied alfalfa crop, the mask of their external borders was done. According to this mask, the cut of part of the calculated *ETa* and its components, being relevant to the investigated areas of alfalfa crops, was made. Those layers were used to find the average daily flows of *ETa* and its components across the investigated areas of alfalfa crops.

#### 2.2.2. Harvesting Data Sets

Assessment of the biological productivity of irrigated alfalfa (*Medicago sativa L, Uzen* variety) in the conducted investigations was based on the average values of the yield of organic carbon, gained for each of three harvests. The samples of harvested alfalfa were collected in a quantity needed for obtaining statistically representative values. The average values of organic carbon yield for three harvests were obtained by standard laboratory processing using mass value of the collected samples, its humidity, and contents of organic carbon in dried biomass.

#### 2.2.3. Irrigation Regime Data Sets

The calculation of the volumes of irrigation water applied to each studied crop was carried out according to the data of the control equipment installed on the water supply infrastructure. Along this infrastructure water from open canals was delivered via pumps to the center pivot sprinkling machines, which operate water supply to the handling fields. As a result, the course of irrigation water, delivered for a specific irrigation water application, was determined as the ratio of volume, delivered to the relevant field, to its area. The total value of the whole amount of irrigation water for the period of irrigation was found by summarizing the relevant amount of each specific water application.

#### 2.2.4. Time Series of ETa and Its Components

In this study, a SEBAL model [63] with FAO 56 technique [64] was used to blend time series *ETa* and its components from 1 May 2012 to 11 August 2012 from radiometer MODIS/EOS Terra and Aqua. Detailed descriptions of the modeling process were described previously [59]. For this purpose, the computer code, based on the description given in [53], was elaborated. Testing of the elaborated computer code showed good reproduction of results given in [51,65,66,67,68,69,70]. The input variables of the code, elaborated on the SEBAL basis, are temperature and air humidity, atmospheric pressure, rainfall, cloudiness, wind speed, and total solar radiation. This model is based on the surface energy budget equation containing in the right part the net radiation flux, the soil heat flux, and the sensible heat flux to the air. Values of these variables were gained from the data of the standard three-hour observations at the meteorological station of the town of Marks. The processing code [71] of the SEBAL model [53] was used to calculate spectral radiance in the visible, near-infrared and thermal infrared part of the spectrum to calculate instantaneous *ETa* and its components for each pixel of MODIS images.

**Figure 3:**
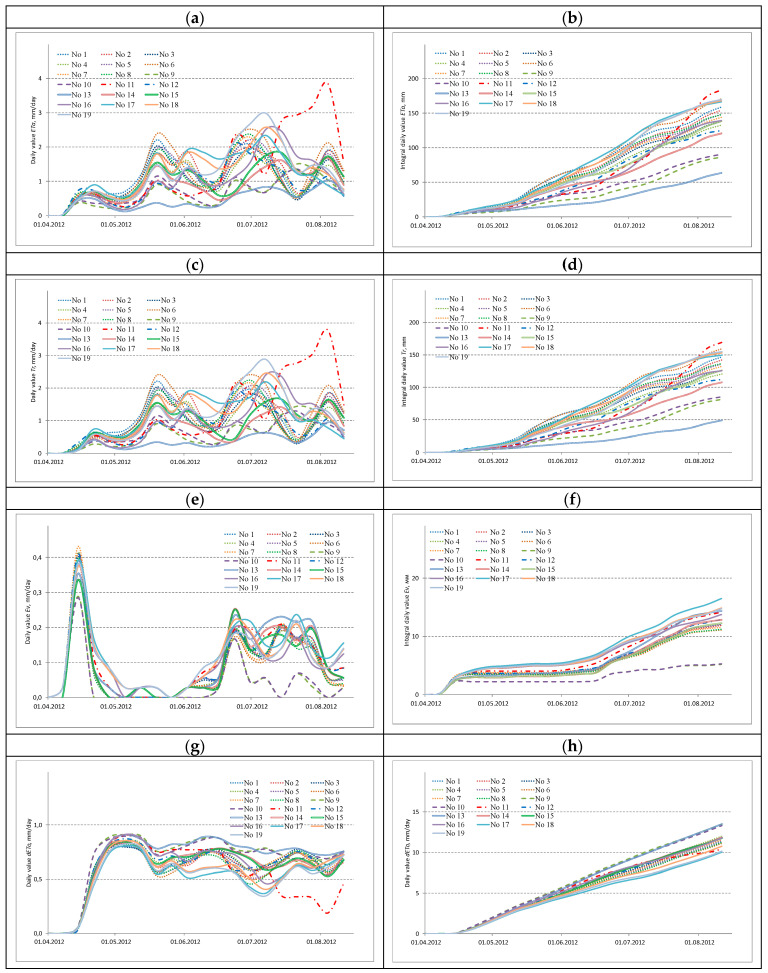
The time series of irrigated alfalfa crops calculated by the SEBAL model: (**a**) daily values of *ETa*; (**b**) integral daily values of *ETa*; (**c**) daily values of *Tr*; (**d**) integral daily values of *Tr*; (**e**) daily values of *Ev*; (**f**) integral daily values of *Ev*; (**g**) daily values of *dETo*; (**h**) integral daily values of *dETo*.

As a result of calculations by SEBAL model, a dataset containing daily values of *ETa* and its components (crop transpiration—*Tr* and evaporation from soil cover—*Ev*) was obtained. These time series, averaged for the convenience of their presentation and analysis, are shown in Figure 3a,c,e, respectively. Next to them, in Figure 3b,d,f, the integral series of the daily fluxes *ETa*, *Tr*, and *Ev* are presented, calculated by successive summation of the values of the members of the daily series, starting from the first.

To compare the series of daily values of *ETa*, *Tr* and *Ev*, their graphical representations were used, shown in Figure 3. For convenient visual identification of their belonging to the corresponding owner, 5 types of lines were used. This allowed visual analyses of the patterns of the presented temporal profiles, associated both with the characteristic group properties and their individual features.

The graphical representation of the series of daily values of *ETa*, *Tr*, *Ev* and *dETo* presented in Figure 3a,c,e,g has a wave-like character, the maxima of which are confined to the moments of irrigation and precipitation. The 1st wave, inherent in the series of all daily values of *ETa*, *Tr*, *Ev* and *dETo* in the second half of March, corresponds to the vegetation period after the snow cover has melted. Its interesting feature is the high values of the series of daily *Ev*, which turned out to be significantly higher than similar values in subsequent periods of vegetation. The 3rd wave, corresponding to the second half of May, falls on the period of the first irrigation which took place almost simultaneously on all crops. The next 2nd wave with slightly lower daily values of *ETa*, *Tr*, *Ev* and *dETo*, corresponding to the beginning of June, can be traced only for crops located on the first (No. 2, No. 4, No. 7 and No. 8) and second (No. 16–19) study sites. This finds its explanation for a series of small precipitations recorded at the weather station in the city of Marks and the corresponding rain front, apparently, passed in a narrow strip along the river. Volga is quite typical in the Saratov Trans-Volga region for this period of the year. As a result of such a passage of this front, precipitation fell on the crops noted above, and those crops that were located at a greater distance from the Volga River were not affected.

The 4^th^ wave, attributable to the period of late June and early July, is fairly consistent. The exception is crops No. 11 and No. 12 of the first, as well as No. 13 and No. 14 of the second production plots, in which this wave falls on earlier dates. In addition, the last 5^th^ wave, which falls on the period of late July and early August, has a fairly consistent nature of crops on the first and second production sites, with somewhat smaller maximums on the latter. At the third study site it falls on slightly earlier dates, which was associated with earlier irrigation.

The analysis of the graphs of daily values of *ETa*, *Tr*, *Ev* and *dETo* showed the similarity within the group of 8 crops (No. 1–8) owned by “Trudovoy”, as well as within the group of 2 crops (No. 9–10) owned by “Berezovskoye”. Obviously, the noted similarity is associated with almost simultaneous irrigation with the same rates. A comparable intra-group similarity can be traced for a group of 2 crops (No. 11–12), which belonged to Volga from the beginning of the growing season to the 3^rd^ decade of June. Since the beginning of July, after the intensification of irrigation of sowing No. 11, this led to a violation of the indicated similarity. In the case of 3 crops (No. 13–15) that belonged to “Meliorator”, a group similarity was noted between the first two, which significantly differed from the last. This is due to the implementation of the irrigation regime on sowing No. 15, with a similar frequency, but with lower irrigation rates. Group similarity can also be traced for all 4 crops (No. 16–19), which belonged to VolzhNIIGiM. However, the irrigation regime of sowing No. 19 in mid-July was intensified by the additional irrigation water application. In turn, the integral series of daily values of *ETa*, *Tr*, *Ev* and *dETo* have a fairly similar form, with the exception of crops No. 15, as well as two crops No. 9 and No. 10, which had significantly lower values during the entire monitoring period in comparison with the rest. Circumstances related to the first, associated with lower irrigation rates, are discussed above. The circumstances associated with the last two are the low quality of irrigation, which led to significant losses of irrigation water to soil and ground runoff, which was noted at the stage of ground surveys and thus was confirmed.

### 2.3. Indicators of Water Efficiency of Irrigation

For a quantitative assessment of the water efficiency of irrigation of alfalfa crops in the Saratov Trans-Volga region, a number of indicators of the third above mentioned group were used, based on ground and space monitoring data sets. The starting point of these indicators is the so-called irrigation water productivity (*IWP*) of agrophytocenosis [41,42,43], which is the ratio of yield *Y* to the total volume of irrigation water *Ir* for the irrigation period.

(1) $$IWP_{Ir}=Y/Ir,$$

The water productivity indicator *IWP_Ir_* is the specific value of yield obtained per unit volume of the irrigation rate. However, in the case of formation of surface or soil-ground runoff, part of the irrigation water is not used for the growth and development of agrophytocenoses, so the values of this indicator will not reflect this kind of inefficiency. In this regard, in order to take into account inefficient losses of irrigation water when it is supplied to an irrigated field, it was proposed to use the volume of total evaporation for the irrigation/vegetation period *ETa* instead of the volume of irrigation water in the denominator of expression (1) [10,18,41,42,43,44,45,46,47].

Further, a definition formulated in works [45,46,47] is applied as an indicator of the efficiency of soil water use by agricultural crops—Water Use Efficiency (*WUE*) that was used for the efficiency assessment in works [19,48] that is presented by the following expression:

(2) $$IWP_{ET}=Y/ET_{a},$$

Both of the above-mentioned efficiency indicators have the same structure, consisting of the ratio of two physically measured parameters. One of these indicators, which is in numerator, is mostly connected with the agronomic aspects of agriculture and plant-growing and presents the mass of either the collected above-ground part of yield or collected above-ground biomass per the unit of agricultural crop area. The second indicator, which is in denominator, is mainly connected with the agrometeorological and agrohydrological aspects of growth and development of the agricultural crop, and also with the applied technologies of agriculture and irrigation.

Therefore, in equation (1) *WUE* indicator expresses the volume of total evaporation from the surface of agricultural crop over the vegetation period, and in equation (2) *WP* expresses only part of the volume of total evaporation, transpirated directly from the crop layer.

The use of both of the above stated indicators allows us to obtain integral assessment of the use of soil water storages by agricultural crops over a vegetation period. It is possible to use the same indicators to assess the efficiency of irrigation performance, but within the framework of a number of limitations and conditions. It is caused by the fact that the volume of water used from the root zone often incorporates not only the volume connected with keeping in this zone of irrigation water infiltrated due to irrigation, but also the volume of water kept in this zone due to spring snow melting and precipitation when the latter fell in the spring period and in the period of vegetation.

Some limitations of both indicators depend of the methods of physical parameters measurements. This remark relates to the methods of measurements of crop yield and above-ground part of biomass as well as to the methods of measurement of the volumes of evaporation and transpiration that are discussed below.

To use indicator (2) with data of evaporation and transpiration of crops some methods based on the Remote Sensing were proposed [49,50], including infrared thermal imagery data by the MODIS sensor [51,52]. In a continuation of the development of methods for processing the results of space monitoring, models were developed that allow dividing the total evaporation flux *ETa* into the fluxes of both its components of evaporation from the surface of the soil cover *Ev* and transpiration of the vegetation cover [53]. This made it possible to use the corresponding results to take into account the unproductive losses of irrigation water associated with evaporation from the surface layer of the soil cover. For this, in the denominator of expression (2), instead of the total evaporation volume *ETa* it was proposed to use the total transpiration volume *Tr* [19,48], which is reflected in the following expression:

(3) $$IWP_{Tr}=Y/Tr_{a},$$

The advantage of IWUE indicator for assessing irrigation efficiency is the close to linear relationship between crop transpiration and their productivity [54]. In addition, the possibility to gain of the value of this characteristic for the agricultural crop space using modern Remote Sensing technologies during the whole period of irrigation also matters.

Thus, due to *IWUE* indicator, assessment of the irrigation efficiency will include individual features of each specific agricultural crop with vegetation characteristics, inherent to this crop for a certain year and formed as a result of realization of the irrigation regime at the background of existing meteorological conditions, as well as realized technological measures (soil preparation, sowing, protection against diseases, addition of fertilizing, etc.) in combination with the characteristics of the soil-ground stratum (nutrients, toxic salts, organic matter, ground water level, etc.), characteristics of the relief of the land surface (presence of micro- and macro-lowerings, aspects of slopes).

This indicator can be used for the comparison at the local level of an irrigation system or irrigation systems situated in the vicinity.

Calculation of the *IWUE* indicator directly for the irrigation period is one of the conditions of its adequate use for the assessment of irrigation efficiency. Irrigation period starts directly from the moment of realization of the first irrigation application. Usually, it coincides with the moment when the water reserves in the root habitable layer left from the spring snow melting and spring irrigation (autumn–spring off-season irrigation) are mostly spent due to evaporation. The end of irrigation period (precisely, its impact on the crop growth and development) occurs after a few days following realization of the last irrigation.

Along with the use of the above three indicators of water productivity of irrigation of agrophytocenosis, indicators of irrigation water efficiency (*IWE*) of agrophytocenoses have been proposed in recent years [17,38]. The impetus for this was the uncertainties associated with yield estimates, both at the level of an individual agrophytocenosis [54], as well as yield uncertainty at the level of individual quasi-homogeneous contours of a separate agrophytocenosis [55,56], during the transition to the precision farming paradigm [57]. As a result, a number of indicators of water efficiency have been proposed, one of which is the ratio of evaporative volume to the volume of irrigation water supplied [58].

(4) $$IWE_{ET}=ET_{a}/Ir,$$

In the denominator of the following indicator, instead of the volume of total evaporation *ETa* a transpiration volume Tr is used [18,31,51].

(5) $$IWE_{Tr}=Tr_{a}/Ir,$$

It is interesting to note that the ratios of pairs of water productivity indicators (1)–(2) (2)–(3) and (1)–(3) are interconnected by two indicators of water efficiency, and in essence all the above five indicators are integral estimates of both water productivity and water efficiency for the considered time period. This does not allow using them to compare individual agrophytocenoses of implemented similar irrigation regimes, in terms of the effectiveness of the water regimes of the root layer.

To overcome this limitation in [59], the combination of SWAP agrohydrological model [60] and SEBAL [53] models was used to simulate the water regime of the same irrigated alfalfa crops. As a result, an empirical relationship was found between the water stress accumulated during the irrigation period and the biomass yield. Given the complexity of using this model [61] in this work, we used its analogue, which uses daily values of basal evaporation *Eto* that is called an average deficit of basal evaporation *dETo.*

(6) $$IWE_{dETo}=\sum \frac{ET_{o}-ET_{a}}{ET_{o}},$$

Note that an important feature of the water efficiency indicator (6) in comparison with the above indicators (1)–(5) is that it does not use either yield values or irrigation volume values, data which in some cases are either absent or contain significant uncertainty.

The purpose of the study was to investigate the opportunity to use irrigation efficiency indicator of alfalfa crops and compare it with indicators of water productivity and water use efficiency. The research involved combining experimental data of yield and water accounting of the growing season in the year 2012, and transpiration calculation by SEBAL model based on MODIS data.

## 3. Results

### 3.1. Statistical Analyzing of Gathered Data Sets

The values of data series of *ETa*, *Tr*, *Ev* and *dETo* between the beginning of irrigation period and their end are shown in Figure 3, as well as data sets of Y and Ir that were used for two types of statistical analysis.

#### 3.1.1. Results of the First Part of Statistical Analysis

**Figure 4:**
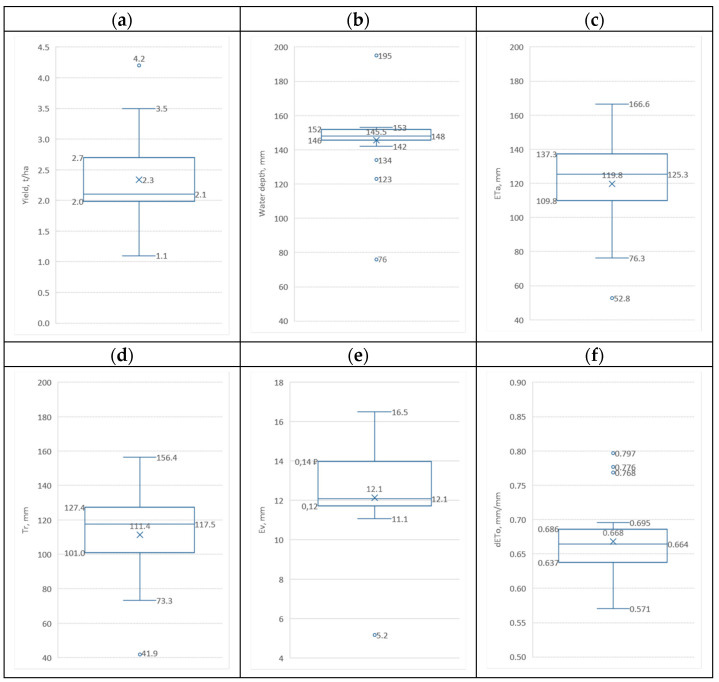
Box and wishers plots showing the lower quartile (Q1), the median (Q2), the upper quartile (Q3), the mean (×), and the minimum and maximum values (◦ outlier values are also depicted) for studied irrigated alfalfa crops during 05.05.2012–10.08.2012 period for: (**a**) yield of organic carbon; (**b**) irrigation depths; (**c**) evapotranspiration; (**d**) plant transpiration; (**e**) evaporation from soil cover; (**f**) average deficit of basal evapotranspiration.

To implement the first of these analyses, the results of the assessment of the statistical parameters of all five datasets presented in Figure 4 were used. The first two of these datasets consisted of data on the yield and irrigation rates of all 19 alfalfa crops. The three following them, corresponding to the integral values of the daily values of *ETa*, *Tr* and *Ev*, were obtained by summing the daily time profiles corresponding to them, starting from the beginning of the irrigation period (12 May 2012) to its end (11 August 2012). The last sixth dataset corresponding to *DETo* was obtained in a similar way, with the values obtained as a result of summation being divided by the number corresponding to the number of days of the selected irrigation period.

Shown in Figure 4a, the statistical yield distribution function Y has an asymmetric character, which is expressed in a significant difference between the values of the average and modal values. This indicates its exponential nature, which is unique to this dataset. There is also one outlier corresponding to crop No. 18 with a maximum yield of 4.2 t/ha. In turn, the statistical distribution function of the irrigation depths *Ir* (Figure 4b) has a narrow bell-shaped form with close values of the average and modal values. This testifies to the predominantly uniform nature of irrigation regimes on 15 of the 19 studied crops. In turn, the remaining four crops had a difference from the noted uniform nature of irrigation regimes, as evidenced by the corresponding outlier values beyond the upper and lower limits of the third quartile, corresponding to crops No. 13 with a minimum irrigation rate of 76 mm, No. 17 (123 mm) and No. 19 (134 mm), as well as for sowing No. 11 with an irrigation rate of 195 mm. The statistical distribution functions of evapotranspiration *ETa*, *Tr* and *Ev*, presented in Figure 4c,d, respectively, are almost symmetrical with mean and modal values without outlier values.

The last function of the relative deficit of basal evapotranspiration *dETo* (weather water demand) presented in Figure 4f, despite the closeness of the values of the average and modal values, has a skew log-normal character, as well as three outlier values beyond the upper and lower limits of the third quartile. The largest of these outlier values corresponds to crop No. 13 with a value of 0.797, followed by crop No. 11 with a value of 0.776 and crop No. 12 with a value of 0.768.

#### 3.1.2. Results of the Second Part of Statistical Analysis

**Figure 5:**
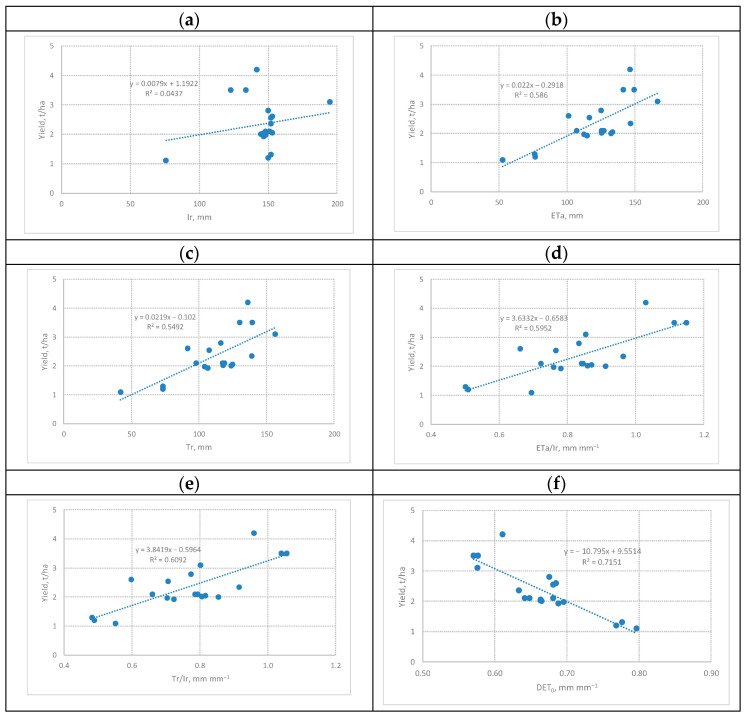
Points and lines showing the values of pares of two datasets and linear correlation functions of studied irrigated alfalfa crops during the period 05 May 2012–10 August 2012 for: (**a**) yield (*Y*) as function of irrigation depth Ir; (**b**) yield (*Y*) as function of evapotranspiration (*ETa*); (**c**) yield (*Y*) as function of transpiration (*Tr*); (**d**) yield (*Y*) as a function evapotranspiration and Irrigation rate (*ETa/Ir*); (**e**) yield (*Y*) as a function transpiration and Irrigation rate (Tr/Ir); (**f**) yield (Y) as a function average deficit of basal evapotranspiration (*dETo*).

The second part of statistical analysis aimed to look for correlations between the collected data sets by finding the parameters of the corresponding linear functions. In graphical form, the results of the correlation analysis are presented in Figure 5.

From the six correlation functions presented in Figure 5, the first one indicates the absence of a significant correlation between yield and irrigation depth (Figure 5a). The five remaining ones show a gradual increase in the values of the coefficient of determination when moving from the function in Figure 5b to the function in Figure 5e. As a result, the best correlation with the yield dataset was demonstrated by the weather demand (*dETo*) dataset; when finding the values, only data obtained from ground-based meteorological sensors, as well as from space-based sensors, were used.

### 3.2. Results of Assessment of Water Efficiency of Irrigation Indicators

Total values of transpiration were used for calculation of the indices of the water use efficiency, water productivity and irrigation efficiency.

Evaluation of the irrigation efficiency of 19 alfalfa crops of the 2nd year of vegetation was carried out using expressions (1)–(6), with data on yield and irrigation rates as well as calculation data for the series of daily values of ETa and Tr, and as relative deficits of basal evaporation *DETo* for the irrigation period (15 May 2012–15 August 2012). Table 1 presents the series of indicator values (1)–(6), as well as the corresponding results of ranking the values of these indicators in descending order.

Thus, calculations of the relevant indices of efficiency by ratios 1–6 were made, and results are presented in Table 1. The ranking of the each of indices obtained was performed for the analysis of the indices’ values. Ranking comprised the fission of series by index descending and assigning a number to each crop relevant to its number in such a series. The results of crops ranking according to the values used under the analysis of the efficiency of indicators are presented in Table 1.

**Figure 6:**
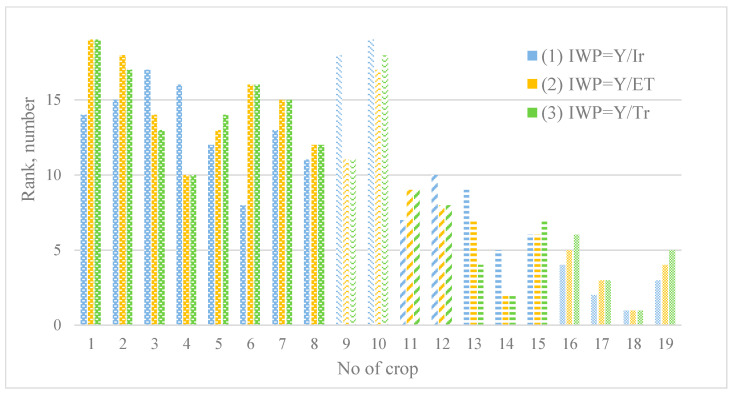
Results of ranking 19 crops of irrigated alfalfa in terms of water productivity (1)–(3).

For the convenience of the analysis processing and for the comparison of the results of ranking of irrigated alfalfa crops on the water use efficiency, the relevant results are presented in Figure 6.

In graphical form, the results of ranking 19 crops of irrigated alfalfa in terms of water productivity (1)–(3) are shown in Figure 4, and in terms of water efficiency (4)–(6) are shown in Figure 6.

#### 3.2.1. Ranking of the Values Indicators of Water Productivity

Comparison of three series of water productivity indicators shown in Figure 6, in which the difference between pairs of corresponding ranks does not exceed the threshold of three values, shows the comparability of the ranks obtained with their help. In the case of six crops No. 1, No. 2, No. 4, No. 6, No. 9 and No. 13, the corresponding rank differences obtained by expression (1), on the one hand, and, on the other hand, by expressions (2) and (3), exceed the specified threshold. Obviously, the significant difference in ranks noted in these cases is a consequence of the inconsistency of the linear correlation between, on the one hand, the data on yield, and, on the other hand, the data on the total volumes of *ETa* and *Tr* during the growing season. At the same time, the first four of these seven indicated crops belonged to the same owner, who provided data on their yield, which could affect the results obtained.

At the same time, in the selected group of six crops, attention is drawn to the high difference in the ranks of two other crops (No. 9 and No. 13), which belonged to different owners, for which low ranks in terms of (1) are adjacent to the average ranks in terms of indicators (2) and (3) in the case of seeding No. 9, and also high in the case of sowing seeding No. 13. The obvious reasons in the case of crop No. 9 are the low values of the total volumes of *ETa* and *Tr* during the growing season, which were the result of large losses of storm water to the soil and ground runoff noted above. In the case of sowing No. 13, the combination of a low rank in terms of indicator (1) and high ranks in terms of indicators (2) and (3) was a consequence of the irrigation regime noted above with low economical rates, which, however, did not allow obtaining a comparable return in type of yield.

#### 3.2.2. Ranking of the Values of Indicators of Water Use Effectiveness

**Figure 7:**
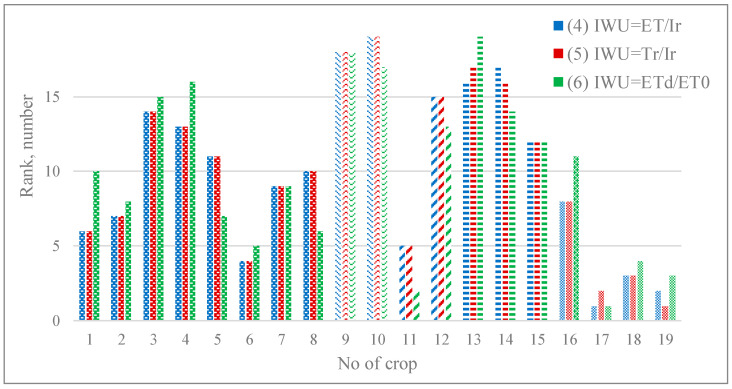
Results of ranking 19 crops of irrigated alfalfa in terms of water efficiency (4)–(6).

Analysis of the differences in the ranks of the second group of water efficiency indicators (4)–(6), presented in Figure 7, revealed two crops No. 11 and No. 15, the difference in ranks of which exceeded the established threshold of three values for pairs of indicators (4)–(6) and (5)–(6). In general, this indicates a fairly close mutual correspondence of these indicators in relation to the studied crops.

As for the noted sowing No. 11, its irrigation regime in the period that began after the 2nd irrigation and included an additional 4th irrigation, led, as noted above, to the formation of the dynamics of *ETa* and *Tr*, which significantly differed from others. As a result, the largest values of the total volumes of both of these flows among all those studied, obtained at this sowing, led to its ranking in terms of indicators (1)–(5) at an average level. However, a more intensive irrigation regime created after the second irrigation resulted in low basal evaporation deficits. As a result, this led to this crop getting a high rank in terms of (6).

For the analysis of crop No. 15, a series of daily values of *Ev* was used, which are unproductive losses of irrigation water for evaporation. The series corresponding to it during the 2nd irrigation water application and after it has the highest values among the others. Obviously, this circumstance led to a decrease in the ranks of indicators of the second group of indicators in relation to the ranks of the first, as well as a decrease in the rank of the indicator (6) in relation to the other two ranks of indicators of the second group.

## 4. Discussion

Irrigation efficiency indicators are introduced to give a posteriori estimate of the decisions made on the irrigation of agrophytocenoses. The diversity of these solutions is quite significant. In many cases, they include criteria to maximize the economic impact of obtaining higher yields per unit of irrigation water used for irrigation. In this case, the use of the water productivity indicator (1), which includes the ratio of both of these values, seems rational. However, the use of this indicator in conditions of scarcity of water resources allocated for irrigation raises legitimate objections [72,73]. This is explained by the analysis of the articles of the water root layer of the soil, which contains three main expenditure articles: (1) transpiration of the vegetation cover *Tr*; (2) evaporation from the surface of the soil cover *Ev*; and (3) deep percolation *Dp*. Of these three components, only the first is usually considered as necessary for the growth and development of agrophytocenoses. In this context, the other two components are considered as losses of irrigation water or, in other words, integral production waste in the cultivation of agricultural plants. These losses depend on many factors related to the characteristics of the soil cover, applied irrigation technologies, as well as irrigation regimes. As a result, the relationship between yield and irrigation water volume is non-linear [74]. The corresponding result was also obtained in this work when analyzing the indicators of water productivity of 19 crops of irrigated alfalfa.

To obtain indicators that allow assessing the effectiveness of irrigation in the framework of management in conditions of water scarcity, other approaches are proposed. A number of them are based on the assessment of water productivity by replacing the volume of irrigation water used for irrigation, depending on the available data, or by the sum of the first two expenditure items, called the actual evapotranspiration *ETa* (2), or only the first of them *Tr* (3). The objectivity of such a replacement is based on numerous experimental data showing the presence of a linear relationship between the yield and the volume of *ETa* or *Tr* during the irrigation period [74,75]. However, the value of crop yield depends not only on the volume of irrigation water used, but also on the applied farming and agrophytocenosis management technologies, as well as the quality of their implementation. As a result, these and a number of other factors lead to a number of uncertainties, leading to a low level of reliability of this linear relationship. This was also demonstrated in the present work using the series of daily values of *ETa* and *Tr* calculated from ground-based and space monitoring data.

One of the directions for the development of methods that use irrigation efficiency as appropriate indicators is based on the use of ratios of expenditure and income items of the water balance (4) and (5). However, the absence in them of a clear connection with productivity requires additional research. One of the approaches to objectify the implicit relationship between *ETa* and *Tr* and yield is based on the use of the *ET* deficit. Thus, this makes it possible to implicitly relate the reduction in yield in relation to the potential, obtained as a result of the implementation of an irrigation regime different from the required one [76]. In the development of this approach to assess the irrigation efficiency, an indicator was proposed based on the use of the integral value of the daily deficits of basal evaporation ETo for the irrigation period. The correlation relationship obtained in this case had a more significant level of reliability of a linear relationship with yield.

## 5. Conclusions

The results of the research presented in this paper demonstrate an opportunity to integrate instrumentally measured data sets to assess the effectiveness of irrigation of alfalfa crops. These sets include Remote Sensing data of vegetation cover characteristics, ground meteorological data of weather conditions, and data of instrumentally controlled water delivered for crop irrigation. These allow us to come to the conclusion of the opportunity to use the irrigation efficiency indicator based on remote sensing data along with the other indicators already used. The expediency of the indicator of the irrigation efficiency use for irrigated agricultural crops is based on the possibility to obtain needed data sets that do not include the crop yield, which is often a difficult practical task in large-scale crops.

Development of new methods and their introduction into the practice of irrigated agriculture will promote realization of policy aimed at the preparedness of the branch for the target-directed overcoming of the probable risks of the incidents of the water resources shortages for irrigation. Realization of this policy will allow us to minimize losses of irrigation water due to the use and application of the new high-effective technologies of irrigation, adopted for the regional and local conditions (both existing and prospective) of the performance of irrigates agriculture.

## Figures and Tables

**Table 1:** Crops ranking according to the values used under the analysis of the efficiency of indicators.

Crop	Water Productivity						Water Efficiency					
	$100*IWP_{Ir}$, t/mm	Rank by $IWP_{Ir}$	$IWP_{ET}$, mm/mm	Rank by $IWP_{ET}$	$IWP_{Tr}$, mm/mm	Rank by $IWP_{Tr}$	$IWE_{ET}$, mm/mm	Rank by $IWE_{ET}$	$IWE_{Tr}$, mm/mm	Rank by $IWE_{Tr}$	$IWE_{dETo}$, mm/mm	Rank by $IWE_{dET}$
1	1.38	14	1.42	19	1.51	19	0.81	6	0.72	6	0.664	10
2	1.34	15	1.47	18	1.57	17	0.79	7	0.71	7	0.663	8
3	1.31	17	1.57	14	1.69	13	0.69	14	0.60	14	0.688	15
4	1.33	16	1.68	10	1.81	10	0.69	13	0.61	13	0.695	16
5	1.39	12	1.57	13	1.68	14	0.75	11	0.67	11	0.648	7
6	1.55	8	1.52	16	1.60	16	0.86	4	0.78	4	0.633	5
7	1.38	13	1.55	15	1.65	15	0.77	9	0.69	9	0.664	9
8	1.42	11	1.60	12	1.70	12	0.76	10	0.68	10	0.641	6
9	0.86	18	1.65	11	1.72	11	0.46	18	0.43	18	0.776	18
10	0.80	19	1.50	17	1.56	18	0.46	19	0.42	19	0.768	17
11	1.59	7	1.82	9	1.94	9	0.82	5	0.74	5	0.576	2
12	1.42	10	1.91	8	2.08	8	0.68	15	0.59	15	0.681	13
13	1.45	9	2.02	7	2.54	4	0.66	16	0.47	17	0.797	19
14	1.70	5	2.43	2	2.68	2	0.60	17	0.51	16	0.684	14
15	1.68	6	2.07	6	2.24	7	0.70	12	0.61	12	0.681	12
16	1.87	4	2.19	5	2.36	6	0.79	8	0.70	8	0.675	11
17	2.85	2	2.11	3	2.57	3	1.05	1	0.91	2	0.571	1
18	2.96	1	2.50	1	2.96	1	0.94	3	0.84	3	0.611	4
19	2.61	3	2.07	4	2.43	5	1.04	2	0.93	1	0.577	3

## Data Availability

The R code, datasets generated during and/or analyzed during the current study are available from the corresponding author on reasonable request. Source data is contained within the article.
